# Epigallocatechin Gallate as a Potential Therapeutic Agent in Endometriosis: A Narrative Review

**DOI:** 10.3390/nu17132068

**Published:** 2025-06-21

**Authors:** Anna Markowska, Zbigniew Kojs, Michał Antoszczak, Janina Markowska, Adam Huczyński

**Affiliations:** 1 Department of Perinatology, Poznań University of Medical Sciences, Polna 33, 60-535 Poznań, Poland; 2 Department of Gynecology and Obstetrics with Gynecologic Oncology, Ludwik Rydygier Memorial Specialized Hospital, Złotej Jesieni 1, 31-626 Kraków, Poland; 3 Department of Medical Chemistry, Faculty of Chemistry, Adam Mickiewicz University, Uniwersytetu Poznańskiego 8, 61-614 Poznań, Poland; 4 Gynecological Oncology Center, Poznańska 58A, 60-850 Poznań, Poland

**Keywords:** endometriosis, green tea, epigallocatechin-3-gallate, EGCG octaacetate, prodrugs, nanoparticles, in vitro, in vivo, clinical trials

## Abstract

Endometriosis is a chronic, hormone-dependent disease that affects women of reproductive age. It leads to numerous adverse clinical symptoms, which significantly impact women’s quality of life. The chronic nature of the disease and its recurrence are the main reasons for the search for new, non-hormonal drugs and drug candidates, either as adjunct treatment options or alternative therapies. The catechin found in green tea, epigallocatechin gallate (EGCG), has been shown to exhibit a wide array of biological activities, which may also contribute to its potential effectiveness in treating endometriosis. The poor physicochemical stability and relatively low bioavailability of EGCG have stimulated the development of a peracetylated prodrug (pro-EGCG) and other solutions, based on nanotechnology, that would eliminate the problems with EGCG. In this review article, we summarize the studies on the effects of EGCG, pro-EGCG, and EGCG-based nanoparticles on the course of endometriosis published in the GoogleScholar and PubMed databases. Of note is the fact that the results of in vitro and animal model studies have suggested that EGCG and pro-EGCG can reduce the number of endometriosis foci and their size and volume, and they can prevent fibrosis by affecting multiple molecular factors and signaling pathways. The promising results provide a basis for using green herbal extracts for endometriosis treatment in a clinical trial. Nevertheless, it should be emphasized that the number of studies on the topic is currently very limited; further expansion in the coming years is necessary. Broad, well-designed clinical trials are also essential to validate the true potential of EGCG and related compounds in the fight against endometriosis.

## 1. Introduction

**Figure 1:**
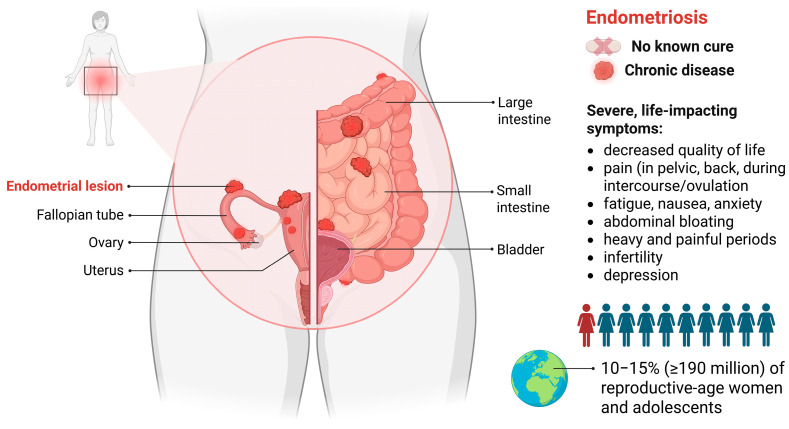
Location and key facts on endometriosis. The figure was created with BioRender.com, accessed on 9 May 2025.

Endometriosis is a benign, chronic, hormone-dependent disease characterized by endometrium-like tissue appearance outside the uterine cavity. It affects 10–15% (≥190 million) of reproductive-age women and adolescents [1,2,3,4] and produces a range of diverse symptoms. The most common ones are painful periods and pelvic pain not associated with menstruation, which are experienced by approximately 70% of women [2], and infertility, which occurs in 30–50% of women [3], ultimately leading to a decreased quality of life. The foci of endometriosis can be located in the ovaries (endometrioid cysts) or peritoneum, or they may deeply infiltrate pelvic tissues, often causing bowel and bladder dysfunction (Figure 1) [1,3].

The etiology of endometriosis is complex and far from fully understood. The hypothesis of retrograde menstruation, first proposed by Sampson, suggests a role of endometrial stem/progenitor cells in the disease’s development [3,5,6]. Cousins et al. [3] presented the results of somatic mutations in endometriosis foci and the endometrium of women with this condition. Among the 40 mapped genes in genome-wide association studies, the most common mutations were identified in the protooncogenes *KRAS* and *PIK3CA*, as well as in deeply infiltrating endometriosis in *KRAS* and the suppressor *PTEN* [3]. Kanno et al. [7] identified an association between *KRAS* and *PIK3CA* mutations and endometriosis resistance to progesterone treatment. According to Zondervan et al. [4], gene disorders are associated with a 26% risk of developing endometriosis. Among the various etiologies of endometriosis development, aberrant hypermethylation of the transcription factor *HOXA10*, which is connected to many malignancies, has also been described [6]. Furthermore, it has been demonstrated that miRNA—a class of short RNA biomolecules with a typical length of 19–25 nucleotides [8]—is commonly dysregulated in endometriosis; this primarily concerns the miR-200 family, miR-143, and miR-145 [9].

In addition to genetic markers, immunological and serum markers are also assessed in cases of suspected endometriosis. Parasar et al. [2] summarized potential diagnostic markers of endometriosis, which include proinflammatory cytokines (e.g., IL-1, IL-6, IL-17, TNF-α), steroids and hormones, growth factors (IGF, TGFβ1), adhesion cells (E-cadherin), factors related to angiogenesis and apoptosis, as well as stem cell markers. Among serum markers, CA-125 is often measured, but many experts and practitioners believe that changes in the measured biomarkers are not sensitive and specific enough to be used as diagnostic screening tests [2,10].

The diagnosis of endometriosis primarily relies on ultrasound imaging, including a recent innovative technique with textural analysis conducted through computer-aided diagnostic tools; also, laparoscopy combined with histopathological evaluation plays a crucial role in the diagnosis of the disease [1,2,11]. Unfortunately, the diagnosis and subsequent treatment of endometriosis are often delayed, as indicated by an analysis by De Corte et al. [10] covering the results of 17 publications, which shows that delays in diagnosis can sometimes extend to 7–10 years. Fortunately, numerous international organizations, including the American College of Obstetricians and Gynecologists, the American Society for Reproductive Medicine (ASRM), and the European Society of Human Reproduction and Embryology, have established diagnostic criteria for endometriosis [5,10,12]. The revised ASRM (rASRM) classification of endometriosis is based on findings from invasive procedures such as laparoscopy. This classification is part of a scoring system that takes into account the presence, size, and depth of lesions (less than 1 cm, 1–3 cm, and greater than 3 cm), as well as their location [13]. Another classification system, known as #Enzian, serves as a comprehensive tool for mapping endometriosis. While primarily reliant on laparoscopic findings, the #Enzian classification can also be made on the basis of ultrasound and MRI imaging [14]. Notably, the diagnosis of deeply infiltrating endometriosis achieves a 97% agreement rate with these methods [14].

Early diagnosis of endometriosis is crucial because of the risk of ovarian cancer development in association with this disease [15,16]. A systematic review by Chiaffarino et al. [15] based on the data of 800 patients from 23 studies has shown that the most common histologic subtypes of endometriosis-associated ovarian cancers are clear-cell carcinomas and endometrioid carcinomas. Driva et al. [17] have examined the frequency of genetic mutations related to the activation of the PI3K/AKT/mTOR signaling pathway, which promoted carcinogenesis in 974 samples collected from patients with clear-cell and endometrioid carcinomas. Of note is the fact that the *ARID1A* mutation was identified in 47% of samples and the *PIK3CA* mutation in 43% [17]. It was also found that clear-cell carcinoma was associated with high levels of proinflammatory cytokines (IL-1, IL-6, IL-8, TNF-α) [17]. Taken together, these findings suggest that endometriosis may serve as a precursor to ovarian cancers.

**Figure 2:**
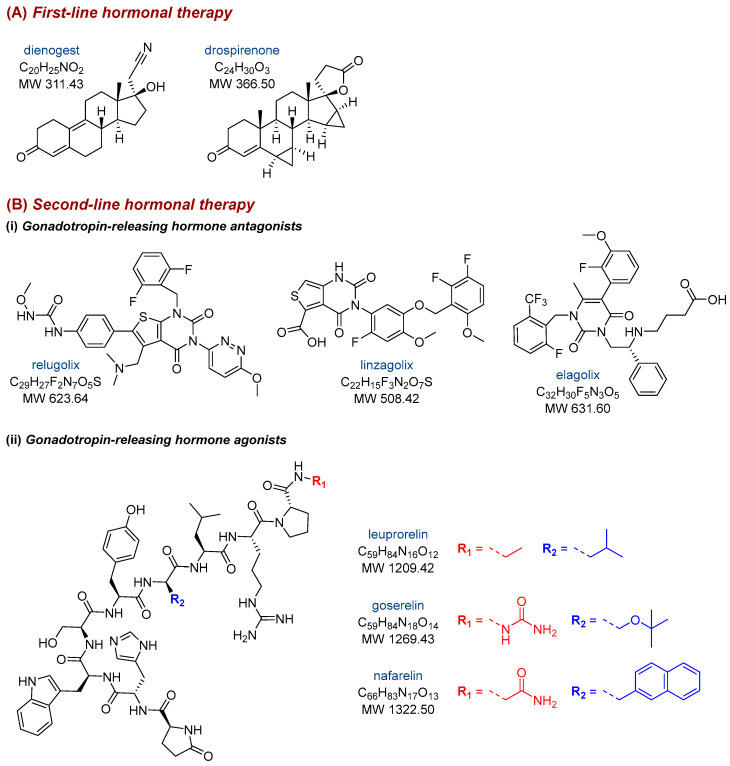
The structure of drugs used in (**A**) first-line or (**B**) second-line hormonal therapy (divided into GnRH antagonists and agonists) of endometriosis.

Treatment of endometriosis includes a wide range of pharmaceuticals, such as birth control pills, progestogens, non-steroidal anti-inflammatory drugs (NSAIDs), and gonadotropin-releasing hormone (GnRH) analogs [1,11,18]. Pharmacological agents can have various effects, including anti-inflammatory action, ovulation inhibition, estradiol level reduction, and modulation of immune functions along with the activity of progesterone receptors. Compounds from the NSAIDs group are commonly used to relieve menstrual pain. For women who are not concerned about immediate fertility, first-line hormonal therapy is typically administered, including contraceptive pills and progestogens, which can be taken orally, as a depot injection, or via a levonorgestrel intrauterine device. The most extensively researched and effective progestogen appears to be dienogest, while drospirenone is one of the recent options available (Figure 2A). If there is no response to first-line therapy, a second-line hormonal treatment may be considered, encompassing GnRH antagonists or agonists. Specifically, GnRH antagonists include relugolix, linzagolix, and elagolix, whereas GnRH agonists are more structurally complex and include leuprorelin, goserelin, and nafarelin (Figure 2B). The role of GnRH antagonists is limited; adverse effects may occur, especially hot flashes and osteoporosis in high-dose cases, and heart disease can rarely occur. To avoid such issues, relugolix combination therapy, containing estradiol and norethisterone acetate, has been developed [1]. Unfortunately, hormonal medications often do not prevent the disease recurrence. On the other hand, Nati et al. [11] presented the results of a 5-year follow-up from nine studies involving 2023 patients who underwent surgery for endometriosis; recurrence occurred in more than 17% of those individuals. It should be stressed that current therapeutic approaches primarily aim to alleviate disease symptoms rather than addressing the underlying mechanisms driving its onset and progression.

Non-hormonal complementary and alternative medications are increasingly being investigated as potential treatments for endometriosis and mitigation of the disease’s consequences [1,19,20,21,22]. Epigallocatechin gallate, a catechin found in green tea, exhibits a wide range of pharmacological activities, which may also contribute to its potential effectiveness in treating endometriosis. Therefore, in this review article, we aimed at gathering information on the effects of catechin on the course of endometriosis based on in vitro and in vivo studies.

## 2. Effects of Epigallocatechin Gallate on Endometriosis

**Figure 3:**
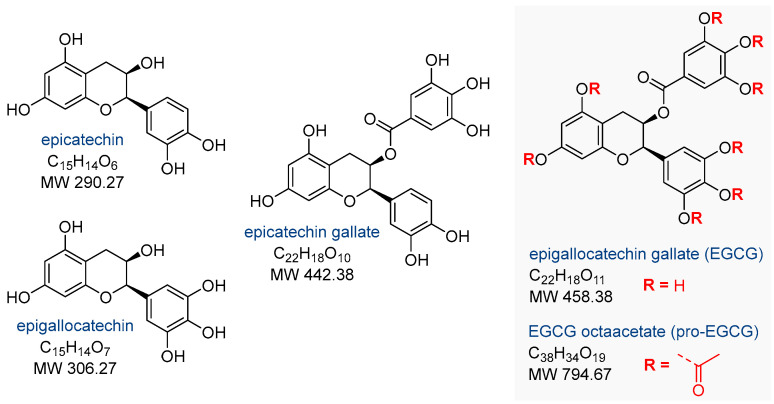
The structure of the four main catechins from green tea and the peracetylated prodrug of epigallocatechin gallate (pro-EGCG).

**Figure 4:**
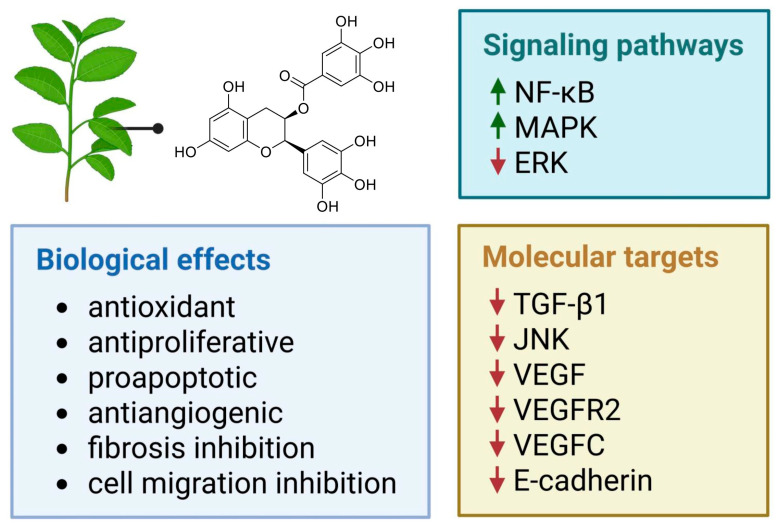
Selected signaling pathways and molecular targets modulated by epigallocatechin gallate, eliciting specific biological effects relevant to the potential treatment of endometriosis. Green arrows—stimulating activity, red arrows—inhibitory activity.

Green tea is derived from the *Camellia sinensis* plant, which originates from East Asia. Its consumption accounts for 20% of the world’s total tea consumption [23]. Green tea contains four major catechins: (–)-epicatechin, (–)-epicatechin gallate, (–)-epigallocatechin, and (–)-epigallocatechin gallate (EGCG) (Figure 3), the latter being its primary bioactive component [23,24,25]. EGCG, by modulating various signaling pathways and molecular targets (Figure 4), exhibits a wide range of biological activities that may help inhibit the development of endometriosis. Notable properties include its antioxidant effects, antiangiogenic activity (by reducing the formation and density of microvessels), antiproliferative activity, stimulation of apoptosis, and inhibition of fibrosis and cell migration (Figure 4) [23,26,27,28,29].

### 2.1. In Vitro and In Vivo Activity of Epigallocatechin Gallate

Several authors have explored the effects of EGCG on endometriosis, both in vitro and in vivo (Table 1). Matsuzaki et al. [29] conducted a study involving tissue samples from 45 reproductive-age patients with deep endometriosis and 10 individuals without endometriosis who were operated on for other gynecologic reasons. The tissue samples were treated with EGCG just before surgery [29]. Additionally, the authors used 40 immunodeficient mice with endometriosis xenografts for in vivo experiments [29]. The tests showed that EGCG significantly inhibits cell proliferation, migration, and invasion and reduces TGF-β1-dependent increases in fibrosis markers, such as ACTA2, type I collagen, and fibronectin [29]. As EGCG effectively prevented the progression of fibrosis associated with endometriosis, the authors concluded that this type of catechin could be a promising candidate for future treatments or prevention strategies for endometriosis [29].

The suppressive effects of EGCG on endometriosis were also evaluated by Ricci et al. [19]. The research involved endometrial cells from 16 women with untreated endometriosis in the proliferative phase, which were obtained through biopsies from the posterior uterine wall [19]. The control group consisted of cells from 15 women treated for infertility and collected during laparoscopy [19]. Additionally, 56 mice, including 27 from the EGCG-treated group, were tested in vivo following the surgical induction of endometriosis [19]. The studies showed that EGCG significantly reduced endometrial epithelial proliferation (*p* < 0.05) and increased apoptosis (*p* < 0.05) [19]. Furthermore, the EGCG-based treatment decreased the number of endometriosis foci and the size of endometrial lesions in the animal models [19]. According to Kamal et al. [23], the levels of NF-κB and MAPK1 mRNA (one of the apoptosis markers) were elevated after EGCG treatment.

Xu et al. [30] transplanted subcutaneously eutopic endometrial tissue from patients with endometriosis into severely immunodeficient mice and then treated the animals intraperitoneally for two weeks with EGCG. Treatment with this type of catechin significantly reduced the size of endometriotic lesions compared to controls (*p* < 0.05) [30]. The glandular epithelium was smaller and showed an eccentric distribution [30]. Angiogenesis in both the lesions and surrounding tissue was underdeveloped, with a significantly lower microvessel size and density (*p* < 0.01) compared to controls [30]. Laschke et al. [31] showed that EGCG substantially inhibits estradiol-induced activation, proliferation, and VEGF expression in endometrial cells in vitro (*p* < 0.05). In vivo, EGCG selectively reduced angiogenesis and blood flow within endometriotic lesions (*p* < 0.05) while leaving vascular development in ovarian follicles unaffected [31]. Histological analysis further confirmed that EGCG treatment leads to the regression of endometriotic tissue [31].

Xu et al. [32] demonstrated that EGCG effectively reduces microvessel formation within endometriotic lesions. This effect was associated with the targeted downregulation of VEGFC and VEGFR2 [32]. The catechin compound disrupted VEGFC/VEGFR2 signaling by modulating several molecular targets, including c-JUN, interferon-γ, MMP9, and the chemokine CXCL3, which are crucial for endothelial cell growth, inflammatory processes, and migration [32]. In endothelial cells, EGCG further reduced VEGFC levels and inhibited the activation of VEGFR2 and ERK signaling pathways [32]. Additionally, EGCG may suppress the progression of endometrial lesions by influencing E-cadherin expression at the cell membrane and decreasing DNA methylation levels in the promoter region of the E-cadherin gene [33]. Using bioinformatic and enzymatic activity analysis, JNK kinase, a member of the MAPK family, was identified as a potential molecular target candidate for endometriosis therapy by EGCG; the catechol product showed inhibitory activity at a nanomolar concentration level (IC_50_ = 94 nM), which was comparable to that of the reference SP600125 compound [34].

A randomized phase II clinical trial was conducted in China involving 185 women aged 18–40 diagnosed with endometriosis who were treated with an oral green herbal extract (NCT02832271). According to the protocol, a daily dose of 800 mg was administered during the 3-month treatment. The disease diagnosis was confirmed by ultrasound imaging, MRI, and biopsy with histopathological evaluation of endometriosis foci at the time of surgery. The clinical trial aimed at evaluating the efficacy and safety of the treatment, but the results have not been posted yet (status as of May 2025). There is no information about the other clinical trials using EGCG in endometriosis, and thus, further studies on the application of catechol in the treatment of endometriosis are highly needed [1,23,26]. According to some investigations, additional bleeding and hypertension in connection with ECGC in endometriosis treatment are not very significant from a clinical perspective [35,36]. On the other hand, it should be noted that an association between the consumption of EGCG and hepatotoxicity has been identified [37].

### 2.2. Activity of the Peracetylated Prodrug of Epigallocatechin Gallate

The octaacetate prodrug of EGCG, known as pro-EGCG (Figure 3), has been synthesized and used to enhance the stability, bioavailability, and biological activity of the native catechin in vivo. Importantly, pro-EGCG showed efficacy in treating endometriosis (Table 2). Wang et al. [38] demonstrated, in an animal model of experimental endometriosis, that pro-EGCG is highly effective after 2 to 4 weeks of usage. The authors found that the size and weight of endometrial lesions and implants were reduced, primarily through the promotion of apoptosis and inhibition of angiogenesis, recommending the use of the EGCG prodrug for endometriosis treatment [38].

Hung et al. [28] conducted in vitro and in vivo studies that revealed the way pro-EGCG targets endometriosis through distinct molecular mechanisms. The research involved endometrial lining cell lines and endometriosis tissue samples obtained from women through laparoscopy or laparotomy, as well as transplanted endometriosis in animal models [28]. The authors were the first to identify that metadherin (*MTDH*), also known as astrocyte-elevated gene 1, which is associated with an increased risk of metastasis in the female reproductive tract, and PX-domain-containing serine-/threonine-like kinase (*PXK*) are significantly overexpressed in women with endometriosis [28]. Of note is the fact that pro-EGCG demonstrated stronger effects on both of these molecular targets compared to unmodified EGCG [28]. By targeting *MTDH*, pro-EGCG inhibited AKT-dependent angiogenesis, and by acting on *PXK*, the prodrug suppressed EGF-dependent angiogenesis, leading to decreased activity of HIF-1α and the VEGF angiogenic pathway [28]. Thus, both *MTDH* and *PXK* represent possible novel targets for the action of pro-EGCG and related compounds. On the other hand, pro-EGCG-based treatment increased apoptosis in cells during in vitro experiments, significantly reduced lesion size by 80% compared to a control group, and effectively prevented the recurrence of endometriosis in vivo [28]. Importantly, pro-EGCG did not affect body weight or disrupt the regulation of hormones such as estrogen and progesterone [28].

A study by Hung et al. [39] identified nicotinamide nucleotide adenylyltransferases NMNAT1 and NMNAT3 as protein targets of pro-EGCG, which was confirmed both in silico and in vitro. In vivo, their expression levels were elevated following pro-EGCG treatment [39]. According to the authors, involvement of bioactive compounds in nicotinate and nicotinamide metabolism may help regulate the redox balance in endometriosis, with pro-EGCG exerting antioxidant effects through interaction with these potential therapeutic targets [39].

### 2.3. Activity of Epigallocatechin-Gallate-Based Nanoparticles

Despite its benefits, the use of EGCG is limited due to the presence of numerous hydroxyl groups in the catechin’s structure, leading to its poor physicochemical stability and low oral bioavailability [26]. According to Talib et al. [37], one reason for EGCG’s low bioavailability is its metabolism in the intestines, which involves the gut microbiome. In this context, nanotechnology may be employed in the treatment procedures to enhance the pharmacological profile and efficacy of EGCG. Although nanoparticles containing EGCG have demonstrated improved stability and bioavailability compared to free catechin in various applications [26], studies focused on the design and synthesis of such systems in the context of endometriosis have been very limited so far.

Singh et al. [40] developed PLGA nanoparticles loaded with a single drug (EGCG or the antioxidant and antiangiogenic doxycycline) and dual-drug-loaded nanoparticles (EGCG plus doxycycline) for the treatment of endometriosis in mice. The dual-drug-loaded nanosystems demonstrated superior efficacy in reducing oxidative stress, angiogenesis, and MMP activity compared to single-drug-loaded ones [40].

### 2.4. Potential Limitations of the Studies

When interpreting the results of the studies, it is crucial to consider their limitations. In vitro cell culture experiments do not fully replicate the complexity of living organisms, including hormonal influences, immune responses, and the tissue microenvironment. These experiments are typically conducted under static conditions, lacking blood flow, mechanical pressure, and cellular interactions. Additionally, they often involve only a single cell type, such as epithelial cells from the human eutopic endometrium [19], and they thus fail to capture the multicellular complexity of the disease. Some studies are also limited by the use of non-human samples and small sample sizes [39].

In vivo studies using rodent models (e.g., mice and hamsters) are commonly employed in preclinical research on endometriosis. However, these models are charged with notable limitations that should be considered when translating findings to humans. Key issues include (i) interspecies differences, (ii) artificially induced disease models, (iii) limited pharmacokinetic relevance, (iv) inadequate representation of clinical symptoms, (v) insufficient modeling of disease heterogeneity, and (vi) short observation periods. Furthermore, the therapeutic promise of EGCG is challenged by its relatively low bioavailability [29].

Mouse models are particularly constrained by the absence of natural menstruation and spontaneous lesion formation [30]. Subcutaneous implantation of human endometrial tissue may also fail to replicate the lesion microenvironment accurately [28]. Although surgically induced models more closely mimic the disease, they only partially reflect human pathology [30]. These models are valuable for studying lesion development and angiogenesis, but assessment of the therapeutic potential of EGCG and other green tea catechins would benefit from more relevant models, such as baboons [41], along with validation through large-scale epidemiological or clinical trials.

In most studies, EGCG and pro-EGCG have been administered via intraperitoneal injection rather than through the oral route [38]. Comprehensive pharmacokinetic data for oral administration are still lacking, and further research is necessary to determine whether these compounds reach endometriotic lesions effectively [38].

Given that endometriosis may be considered an angiogenic disease, antiangiogenic agents like EGCG represent a promising therapeutic approach [31]. However, this strategy also has limitations. Older endometriotic nodules, often fibromuscular and poorly vascularized, may be less responsive to antiangiogenic therapy [42]. Consequently, such treatments may be more effective in preventing new lesion formation than in eliminating existing ones [31]. Postoperative administration of antiangiogenic agents could, however, help prolong pain-free intervals and reduce recurrence [43].

Finally, human-based studies are essential to validate the proposed mechanisms and confirm the antiangiogenic and other therapeutic effects of EGCG and pro-EGCG before clinical application [28]. These compounds may interact with different protein targets depending on the cell type, potentially activating diverse signaling pathways [44,45]. Their mechanisms of action may vary with the cellular context, highlighting the need to explore their effects within the endometriotic microenvironment.

## 3. Literature Search Strategy

We conducted a comprehensive literature search using the PubMed and Google Scholar databases to identify relevant studies related to the topic of epigallocatechin gallate (EGCG) and its potential role in endometriosis therapy. The search was performed using the keywords “epigallocatechin gallate” or “EGCG” in combination with “endometriosis”. Given the known limitations in the bioavailability of native catechins, we also included studies investigating the peracetylated prodrug of EGCG by using the keywords “pro-EGCG” and “endometriosis”. In addition, we manually screened the reference lists of the selected articles to identify further relevant publications that may not have appeared in the initial database search.

## 4. Conclusions

Endometriosis is a chronic disorder characterized by the ectopic presence of endometrial glands and stroma outside the uterine cavity. Affecting approximately 10–15% of women of reproductive age, it presents a significant clinical challenge and is linked to considerable social, sexual, and reproductive burdens. Timely diagnosis and effective treatments of endometriosis are crucial, yet access to them remains limited in many areas, particularly in low- and middle-income countries. Moreover, current treatment options, including analgesics, hormonal therapies, and surgical interventions, are often viewed as suboptimal due to their variable effectiveness, substantial side effects, and a high rate of disease recurrence.

There is no known cure for endometriosis, and its treatment typically focuses on managing symptoms of the disease. Green tea and its predominant bioactive catechin, epigallocatechin gallate (EGCG), exhibit various biological activities, particularly those leading to antiangiogenic, antiproliferative, antimetastatic, and proapoptotic effects. These properties support the potential of EGCG as a therapeutic agent for endometriosis. Preclinical studies, both in vitro and in vivo, have demonstrated that EGCG can reduce the number, size, and volume of endometriotic lesions and inhibit fibrosis by influencing various molecular mechanisms and signaling pathways. To enhance the therapeutic potential of EGCG, particular attention has been directed towards strategies that improve its bioavailability and physicochemical stability. These include the development of EGCG-based prodrugs, such as peracetylated EGCG (pro-EGCG), and the use of EGCG-decorated nanosystems. Additionally, a clinical trial investigating the use of green herbal extracts for endometriosis treatment has been initiated, although its outcomes have not yet been disclosed.

Despite these encouraging results, the existing literature remains inadequate to conclusively determine the efficacy of EGCG in the clinical management of endometriosis. Further comprehensive research, particularly well-structured clinical trials, is necessary to evaluate the potential of EGCG and its derivatives in disease prevention, adjunctive therapy, and relapse mitigation.

## Figures and Tables

**Table 1:** Studies on the effects of epigallocatechin gallate on endometriosis.

In Vitro Model	Animal Model	EGCG Treatment	Main Results	Ref.
Tissue samples from 31 reproductive-aged patients, including 16 patients with untreated endometriosis (stages I and II, as classified by ASRM) and 15 control patients without endometriosis	56 adult (2 months old) female BALB/c mice with endometriosis-like lesions induced via autologous transplantation of one uterine horn to the bowel mesentery	In vitro tests: 0, 20, 40, 80, or 100 µM Animal studies: 20 mg/kg/day or 100 mg/kg/day by esophageal gavage for four weeks	EGCG treatment reduced the mean number and volume of established endometriotic lesions, inhibited cellular proliferation, decreased vascular density, and enhanced apoptotic activity within the lesions. EGCG also reduced proliferation and promoted apoptosis in primary cultures of human endometrial epithelial cells	[19]
Endometrial and endometriotic tissue samples collected from 45 patients (median age: 31.0 years; range: 22–36) with histologically confirmed deep endometriosis and from 10 control patients without endometriosis, including 6 with uterine myomas (median age: 31.5 years; range: 28–34) and 4 with tubal infertility (median age: 29.0 years; range: 26–32)	40 adult (7–8 weeks old) female Swiss nude mice, which received a single injection of proliferative endometrial tissue derived from ten distinct donor samples after the acclimation period	In vitro tests: 50 or 100 µM Animal studies: 50 mg/kg/day by i.p. injections on day 7 or 14 after endometrial tissue implantation and continued for two weeks	EGCG treatment suppressed the proliferation, migration, and invasion of endometrial and endometriotic stromal cells derived from patients with endometriosis. In vivo studies further demonstrated that EGCG attenuated the progression of fibrosis associated with endometriosis	[29]
	Adult (6 weeks old) female SCID mice with eutopic endometrium transplanted from endometriosis patients (stage III, as classified by ASRM)	5 mg/kg/day or 50 mg/kg/day by i.p. injections for two weeks	Endometriotic lesions were smaller after EGCG treatment. Angiogenesis within the lesions and surrounding tissues was underdeveloped, and apoptotic activity within the lesions was increased	[30]
Isolated hamster endometrial stromal and glandular cells	Female Syrian golden hamsters (8–10 weeks old) with endometrial fragments and ovarian follicles transplanted into the dorsal skinfold chambers	In vitro tests: 40 µM Animal studies: 65 mg/kg/day by i.p. injections for three days or two weeks	EGCG suppressed E2-induced activation, proliferation, and VEGF expression in endometrial cells, inhibited angiogenesis and reduced blood perfusion in endometriotic lesions, promoted regression of endometriotic lesions, and downregulated VEGF expression in the eutopic endometrium	[31]
Human microvascular endothelial cells	30 adult (6 weeks old) female mice with endometrium transplanted from endometriosis patients (stage III, as classified by ASRM)	In vitro tests: 10–50 μM Animal studies: 50 mg/kg/day by i.p. injections for three weeks	EGCG exhibited antiangiogenic activity in endometriosis by inhibition of this process and selective suppression of the expression and signaling of VEGFC and its receptor VEGFR2	[32]
	36 adult (6–8 weeks old) female BALB/c mice with transfected endometrial fragments obtained from patients aged 44–52 who had undergone hysterectomy due to ovarian endometriotic cysts and uterine myomas	8.333 mg/mL by i.p. injections every other day over 16 days	EGCG suppressed the growth of endometrial lesions and reduced the expression of E-cadherin	[33]

**Table 2:** Studies on the effects of the peracetylated prodrug of epigallocatechin gallate on endometriosis.

In Vitro Model	Animal Model	Pro-EGCG Treatment	Main Results	Ref.
Human endometrial stromal SHT290 cell line, human endometriotic HS293 (C). T cell line	Adult (8 weeks old) female C57BL/6 mice bearing subcutaneously transplanted endometrial tissue, implanted into subcutaneous pockets located on the abdominal wall of each recipient	In vitro tests: 0–300 μM Animal studies: 25 mg/kg or 50 mg/kg taken orally for four weeks	Pro-EGCG exhibited a more potent inhibitory effect on lesion growth compared to EGCG. The prodrug showed efficacy in suppressing lesion development, enhancing apoptosis within lesions, downregulating the angiogenic marker CD31, and preventing lesion recurrence. Pro-EGCG did not impact body weight or alter endogenous levels of female hormones	[28]
	32 NOD-SCID mice with homologous endometrium subcutaneously transplanted from 8-week-old female CMV-Luc mice	50 mg/kg/day by i.p. injections for four weeks	Pro-EGCG suppressed the development, growth, and angiogenesis of experimental endometriosis in mice, demonstrating superior efficacy, enhanced bioavailability, and stronger antioxidant and antiangiogenic properties compared to native catechin	[38]
Primary human endometrial stromal cells isolated from a single healthy female donor without a diagnosis of endometriosis	Adult (8 weeks old) female C57BL/6 mice with subcutaneously transplanted endometriotic tissues of the uterine fragments from the mouse donor group	In vitro tests: 0–300 μM Animal studies: 50 mg/kg/day by i.p. injections for three weeks	Pro-EGCG upregulated the expression of the NMNAT enzymes	[39]

## Data Availability

Not applicable.
